# Associated Factors and Outcome of Uterine Rupture at Suhul General Hospital, Shire Town, North West Tigray, Ethiopia 2016: A Case-Control Study

**DOI:** 10.1155/2017/8272786

**Published:** 2017-12-18

**Authors:** Tefera Marie Bereka, Amlaku Mulat Aweke, Tewodrose Eshetie Wondie

**Affiliations:** ^1^Midwifery Department, Debre Markos University, College of Medicine and Health Science, Debre Markos, Ethiopia; ^2^Midwifery Department, Bahir Dar University, College of Medicine and Health Sciences, Bahirdar, Ethiopia; ^3^Health Informatics Department, Debre Markos University, College of Medicine and Health Sciences, Debre Markos, Ethiopia

## Abstract

**Background:**

Uterine rupture is tearing of the uterine wall during pregnancy or delivery. It may extend to partial or whole thickness of the uterine wall. It is usually a case where obstetric care is poor. In extensive damage, death of the baby and sometimes even maternal death are evident.

**Objective:**

This study assesses associated factors and outcome of uterine rupture at Suhul General Hospital, Tigray Region, Ethiopia, 2016.

**Methodology:**

A case-control study was conducted by review of data from September 2012 to August 2016. A total of 336 samples were studied after calculating by EPI-INFO using proportion of multiparity (53%) and ratio of 1 : 2 for cases and controls, respectively. Analysis was done using SPSS version 20. Bivariate and multivariate logistic regression was applied with *p* < 0.05.

**Result:**

ANC, grand multiparity, malpresentation, and obstructed labor had association, but previous cesarean delivery was not significant. Perinatal mortality was 105 (93%) versus 13 (5.8%) in cases and controls, respectively. Anemia was highest for both groups (53.7% versus 32.1%).

**Conclusion:**

Majority of uterine rupture is attributed to prolonged or obstructed labor. Cases of uterine rupture had prompt management preventing maternal mortality, but burden of perinatal death is still high.

## 1. Introduction

Maternal and neonatal morbidity and mortality are among standing challenges in the arena of reproductive health. Uterine rupture is one of the contributors of obstetric morbidity and mortality in developing countries [1]. Uterine rupture is tearing of the uterine wall, which can happen any time during pregnancy or delivery. The rupture can take different forms which include a spectrum of problems ranging from asymptomatic disruption to overt uterine rupture. A large rupture may be associated with massive hemorrhage with complete fetal extrusion from the uterus into the peritoneal cavity [2]. And the overall rates for uterine rupture ranged from 0.1% to 1% in countries where obstetric health care is diminished [1].

The incidence of rupture to scarred uterus is relatively common and usually happens after trial of labor in patients with previous cesarean delivery having surgical scar [3]. A countrywide study done in Switzerland in 92 cases of uterine ruptures evidenced that the risk of rupture among women with a trial of labor was double as compared with those who do not have trial of labor [4]. Incidence of uterine rupture which occurs in previously unscarred uterus tends to decline (1 in 5700 to 20,000 pregnancies) with improvement of modern obstetric services [5]. Though the previous study stressed on concern of scarred uterus, other findings from countries such as Nigeria, Ghana, Ethiopia, and Bangladesh indicated that about 75% of cases of uterine rupture were associated with unscarred uterus showing both faces of rupture notably seeking due attention [1].

Ethiopia is among the five countries with high maternal mortality ratio in the world, and 676 maternal deaths per 100,000 live births were reported in 2011 Ethiopian Demographic Health Survey (EDHS) [6]. A review of causes of maternal mortality conducted in Ethiopia, which compares two different decades, clearly depicts the importance and need of attention to rupture of uterus. According to this study, the top four causes for the years 1980–1999 were abortion-related complications (31%), obstructed labor/uterine rupture (29%), sepsis/infection (21%), and hemorrhage (12%). But obstructed labor in combination with uterine rupture had become the leading cause of maternal death accounting for thirty-six percent of deaths followed by hemorrhage (22%), hypertensive disorders of pregnancy (19%), and sepsis/infection (13%) between the years from 2000 to 2012 [7].

Even though indicators for emergency obstetric care dictate that the fatality of any obstetric condition should not exceed 1%, the burden of death due to uterine rupture is still higher in Ethiopia indicating that need for attention is still required for this obstetric sequel [8]. For instance, studies conducted in parts of Ethiopia showed the prevalence was 3.7% at Gimbie Adventist Hospital, West Wollega, Ethiopia, and 2.6% in Shashemene referral hospital, northeast Ethiopia [9, 10].

Fortunately, ruptured uterus is a preventable condition. And to do this, it was essential to determine the associated factors so as to act accordingly. With this view of preventive aspect and its fact as a public concern, the study was conducted with the objective of identifying associated factors and outcome of this problem. And the result presented can have significance in devising appropriate preventive strategies in preventing occurrence and complication of uterine rupture for health professionals and health planner as well.

So, this study was conducted with the objective of identifying associated factors of uterine rupture and assessing maternal and fetal outcomes of uterine rupture in Suhul General Hospital, Tigray Region, Ethiopia.

## 2. Methodology

### 2.1. Study Area and Period

This study was conducted at Suhul General Hospital, Shire town, Tigray Region, Ethiopia, from Nov to Oct 2016 with review data of the five-year period (September 2012 to August 2016). Shire is found at a distance of 1096 km northwest from Addis Ababa, the capital city of Ethiopia. It is one historic town in Ethiopia. Shire, also known as Inda-Silassie, in Tigrigna “house of trinity,” is a town and separate woreda in Tigray region. It is the administrative centre of the *Semien Mirabawi* zone of the Tigray Region. This town has latitude and longitude of 14°6′N 38° 17′ E/14.100 N 38.283°E with altitude of 1953 m above sea level. Based on the 2007 national census conducted by the central statistical agency, this town has total population of 47,284, of whom 21,867 are men and 25,417 women. Majority of the inhabitants (85.11%) follow Ethiopian Orthodox Christianity and Muslims account for 14.67% of the population [11].

Suhul Hospital is a governmental hospital named after “Suhul,” a renowned warrior of the area during the Italo-Ethiopian War. The hospital was established in 2003. It is estimated to serve more than one million people residing in the town and neighboring areas. The hospital is organized in different units, that is, outpatient department, operating theatre (two major operating rooms and one minor operating room), and paediatrics, medical, surgical, and maternity wards providing inpatient service. Maternity unit is the one which gives inpatient and outpatient services. It has 34 beds (4 in waiting room, 30 for Obs/Gyn admission) and 4 beds for delivery service. There are 173 health professionals and of them, three are at the specialty level (one internal medicine specialist, one general surgeon, and one obstetrics and gynecology specialist). There are fourteen midwives working in delivery room both at diploma and BSc level.

### 2.2. Study Design

This study was an unmatched case-control study.

### 2.3. Population

#### 2.3.1. Study Population

All women who gave birth at Suhul Hospital during the study period.

#### 2.3.2. Sample Population

All women who were diagnosed to have uterine rupture at Suhul Hospital and selected women who do not have uterine rupture during the period.

### 2.4. Inclusion and Exclusion Criteria

Mothers who gave birth at Suhul Hospital from September 2012 to August 2016 and diagnosed with uterine rupture were included in the case groups, and those parturients who gave birth and not developed uterine rupture during the period were recruited as controls. Cases of ruptured uterus which occurred following instrumental applications were not included in the study.

### 2.5. Sample Size Determination

Taking proportion of grand multiparity in cases (54.4%) and controls (12.6%), which is an associated factor in the study conducted in Nigeria, sample size was determined using EPI-INFO giving total of 340 samples. Since the total number of uterine rupture which meets the inclusion was 112 cases, control group was determined with a ratio of 1 : 2 ration giving 224 mothers, and total sample size of 336 parturients were reviewed in the study.

### 2.6. Sampling Procedure

Samples were selected among mothers admitted to labor ward of the hospital in the study period of five years, September 2012 to August 2016. All cases of uterine rupture (112) in the study period were extracted from hospital records. Parturient cards that do not have uterine rupture are selected from each year with the ratio of 1 : 2 for cases in respective years. And the controls were first two women who delivered without ruptured uterus immediately after each one with ruptured uterus. The cases were unmatched type, and controls were selected randomly by taking two controls/women admitted without rupture after each case of woman with ruptured uterus.

### 2.7. Data Collection Tool and Procedure

The data were collected using pretested checklist which was prepared in English. The content of this checklist was adopted from different researches, in a way which can address the objectives of this study. The data collectors were four 2nd year MSc in Integrated Emergency Obstetrics and General Surgery (IEmOGS) students with close supervision by the principal investigator.

Secondary data were collected primarily by reviewing parturient cards using a structured checklist. In case information is not fulfilled in the card, the delivery registry book, operation theatre notes, and health management information system (HMIS) unit of the hospital were used to retrieve data.

### 2.8. Data Quality Control

Data collectors were trained for two days to have common understanding of the study. The checklist was explained to the data collectors to avoid unclear points. Pretest of the checklist was conducted on 5% cards in Adigrat hospital.

### 2.9. Study Variables

#### 2.9.1. Dependent Variable

Uterine rupture is the dependent variable of this study.

#### 2.9.2. Independent Variables

Age, residence, parity, ANC follow-up, duration of labor, uterine scar, birth weight, number of gestation, malpresentation, type of onset of labor, prolonged or obstructed labor, and labor intervention are explanatory variables of this study.

### 2.10. Operational Definition

Some of the variables are found differently from literature to literature. So, in this study, the following terms are used:

- ANC follow-up—a woman who had at least one antenatal care in current pregnancy.
- Labor intervention—when progress of labor is intervened with oxytocic drugs or instrumental delivery is applied to shorten duration the labor.
- Management type is medical or surgical intervention done for uterine rupture.
- Onset of labor—how the labor started, that is, either spontaneous onset of labor without intervention or induced labor for obstetric or medical indication.
- Outcome—anything happened to the mother or the fetus prior to and following management of uterine rupture. It includes maternal death, hypotension, shock, infection, hysterectomy, BTL, prolonged hospitalization, fetal death, low Apgar, live birth, and other.
- Spontaneous uterine rupture—defined as rupture without any iatrogenic manipulation, trauma, or any oxytocic drug use.
- Uterine rupture—tearing of the uterus and it can be spontaneous or traumatic.

### 2.11. Data Analysis

The data were entered in EPI data version 7. The analysis was performed using SPSS version 20. Descriptive statistics, that is, frequencies and percentages, were used to report categorical variables using tables and graphs. Bivariate logistic regression was performed to see the association of each independent variable with the outcome variable with 95% CI and significance point *p* < 0.05. Variables with p<0.05 in bivariate analysis were recruited to multivariable logistic regression and association was determined with cut of point P<0.05.

### 2.12. Ethical Consideration

The study was started after securing letter of ethical approval from the ethical review board of Mekelle University College of Health Sciences. The hospital director was officially informed before commencing the data collection. Moreover, the information is used anonymously and merely for this study.

## 3. Result and Discussion

### 3.1. SocioDemographic and Obstetric Characteristics

Among the total samples, majority of mothers (261; 77.7%) were rural residents, and higher cases of uterine rupture (107; 95.5%) were also from rural residents. The age of the mothers ranged from 17 to 43 and 18 to 42 years for cases and controls, respectively, and majority of uterine ruptures (35; 31.2%) occurred in the age group of 25–29 years old. Though grand multiparous accounts for 61 (19.9%) of total sample, the share of rupture in this group was high (36; 32.1%). Among the cases, 46 (41.1%) cases had no ANC follow-up, and only 11 (4.1%) women in control group have no ANC follow-up (Table 1).

To describe rupture of uterus based on the type, majority of the rupture is diagnosed as complete rupture based on extent and is anterior type based on the site of rupture (Table 2).

### 3.2. Factors Associated with Uterine Rupture

Parity, ANC follow-up, interpregnancy interval, labor duration, type of onset labor, obstructed labor, and malpresentation were associated with uterine rupture in bivariate analysis with cut point of *p* < 0.05. But, of them, five variables (Parity, ANC follow-up, duration of labor, obstructed labor, and malpresentation) were found to have significant association in multinomial analysis (Table 3). Even though several studies conducted in Nigeria, Israel, and Ethiopia found a significant association of age of the mother, interdelivery interval, gestational age, fetal weight, and previous cesarean scar, this study showed no significant association [12–14].

The odds of developing uterine rupture in mothers diagnosed with obstructed labor were six times higher than their counter (*p* < 0.05, 5.76 (2.32–14.28)). This finding is in aggregate with findings in many developing countries such as Ethiopia, Uganda, and Nigeria [14–16]. When the total duration of labor is greater than 24 hours, they are 24 times more likely to develop uterine rupture when compared to mothers laboring for less than 12 hours. (*p* < 0.05, AOR = 24.387, CI = 7.914–74.148). This finding is supported by other studies [9, 13, 14]. From this, one can conclude that the impact of prolonged and obstructed labor is still not averted in *shire* town and neighboring area of northwest Ethiopia.

Plenty of literatures and health advocates put forward the importance of ANC follow-up in eradicating maternal morbidity and mortality and achieving safe motherhood. In supporting this, some studies show significant association of ANC follow-up with uterine rupture [3, 15, 17, 18]. This study also showed that when comparing risk of uterine rupture with ANC status, women who do not have ANC follow-up are 15 times more likely to develop uterine rupture than women who have two or more ANC follow-ups (*p* < 0.05, 14.62 (4.55–46.99)). Likewise, women who had only one ANC visit are at 5 times greater risk than women who had two or more visits (*p* < 0.05, 5.380 (2.283–12.682)). The significance of ANC is not surprising because it is contact time for identifying problems such as malpresentation and time to advise on sign of labor and timely arrival of laboring women to health facilities. Besides this, since ANC follow-up is crucial for early identification of malpresentation and other risky pregnancies, it has a pivotal role in averting occurrence and sequel of obstetric-related tragedies including rupture of uterus.

Previous caesarean section was not significant in this study, which was also the finding in the study conducted in Nigeria [18]. This may probably due to the fact that such risky women are closely followed and advised during ANC follow-up, closely monitored during labor, and could have timely intervention prior to occurrence of complication.

### 3.3. Maternal and Fetal Outcome

The perinatal mortality in both case and controls (109 (93.1%) versus 14 (6.0%)) were higher when compared to a study conducted in Israel (19.0% versus 1.4%) [19]. But the result is in hand with plenty of cross-sectional studies conducted in Ethiopia, Ghana, and India [10, 13, 20, 21]. Probably, higher amount of blood loss especially during complete rupture, which is a case in this study (93; 83.1%), which in turn occurred due to discrepancy in health care system, might be responsible for the increased incidence of stillbirth. Nonoccurrence of maternal death among the cases was an important finding, and this might be resulted from the fact that majority of ruptures occurred in a way to referring from primary health facility to the hospital. In such cases, maternal life can be salvaged in aggressive management. So, from this, one can clearly see that neonates are at the verge of death when rupture of uterus occurs.

Women with uterine rupture also cost a lot than their counter. For instance, blood was transfused in 35 (31.2%) cases, but only 7 (3.1%) were transfused among controls. Prolonged hospitalization reaching 8–12 days was one of the problems in 62 (55.5%) mothers among cases, whereas only 3 (1.3%) were stayed for same duration among the control (Table 4).

One of the important outcomes of uterine rupture was loss of fertility following management. Repair of tear was the commonest type of operation (61; 54.5%) (47 with BTL and 14 without BTL). Inline with this finding, a study conducted in Turkey, repair of ruptured uterus was the commonest type (58; 95.1%) and is advisable type of procedure when maternal bleeding is high and duration of operation needs to be shortened [22]. But still rupture of uterus was responsible for loss of fertility in 98 (87.5%) cases following one of the procedures among STH, TAH, or repair with BTL.

**Figure 1:**
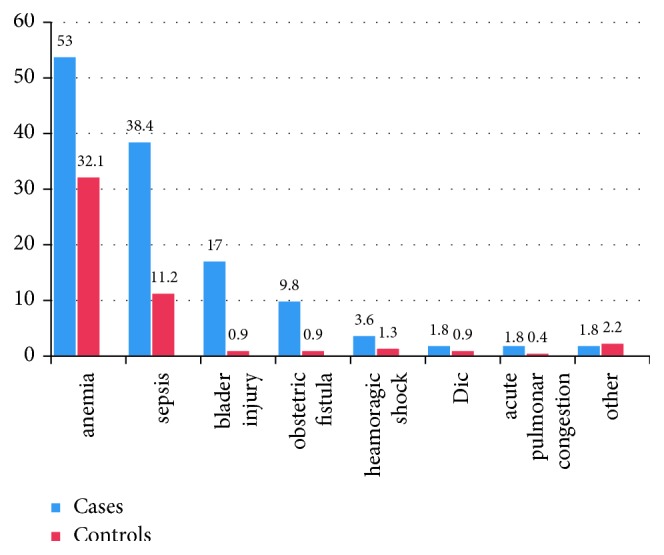
Complications developed in women with uterine rupture and without rupture of uterus in Shire Hospital, North Ethiopia, 2016. ∗ Other complications include urethral ligation, posttrauma psychosis, and foot drop.

Anemia (60; 53.7%) was the commonest complication of uterine rupture in this study. Supporting this finding, anemia was the leading complication in the study conducted in Adigrat and Debre Markos [13, 23]. Since this case-control study showed that anemia was among major complications in the control group (72; 32.1%), and baseline HCT in 79 (70.5%) of cases and 69 (30.1%) of controls were anemic during admission, one can see the occurrence of anemia is not merely attributed to uterine rupture but also the baseline reserve of hemoglobin in woman in the area may be lower (Figure 1).

Sepsis (43; 38.4%) was also among the commonest complications of uterine rupture, which in turn necessiates for the prolongation of hospital stay until it gets treated and increases total cost of treatment as explained by study conducted in Debre Markos general hospital [13]. Eleven (9.8%) of mother with uterine rupture developed obstetric fistula. Even though the occurrence of obstetric fistula is lower than a figure reported in a nearby town at Adigrat hospital (6; 12.5%), the magnitude of obstetric fistula was a big concern in a woman with ruptured uterus than her counterpart (11 (9.8%) versus 2 (0.9)).

## 4. Conclusion and Recommendations

Uterine rupture is a devastating condition that carries grave risks to the newborn and the mother as well. But fatal conditions were burdened to the fetus. Obstructed labor, prolonged labor, malpresentation, absence of poor ANC utilization, and grand multiparity were associated with uterine rupture. And, in fact, all these conditions can be averted by paying attention to proper utilization to one of the factor, antenatal care.

The impact of uterine rupture highly affects the life of the fetus, resulting spectrum of low Apgar score to still birth. And mothers are affected by the problem reaching from immediate complication to long-lasting impacts, such as loss of fertility. For these reasons, coordinated effort must be made at health institutions, in the community level and policy makers with special emphasis for rural areas in enhancement of access and utilization to BEmONC and CEmONC service.

## Figures and Tables

**Table 1:** Sociodemographic and obstetric characteristics of parturient in Suhul General Hospital, Shire Town, North West Tigray, Ethiopia, 2016.

	Cases (*n* = 112)	Control (*n* = 224)	Total (336)
*Residence*			
Urban	5 (4.5%)	70 (31.2%)	75 (22.3%)
Rural	107 (95.5%)	154 (68.8%)	261 (77.7%)
*Age in years*			
<20	6 (5.4%)	11 (4.9%)	17 (5%)
20–24	18 (16.0%)	64 (28.6%)	82 (24.4%)
25–29	35 (31.2%)	59 (26.3%)	94 (28%)
30–34	22 (19.6%)	42 (18.8%)	64 (19%)
35–39	16 (14.3%)	33 (14.7)	49 (14.6%)
>40	15 (13.4%)	15 (6.7%)	30 (8.9%)
*Parity*			
Nullipara	9 (8.0%)	62 (27.7%)	71 (21.1%)
Multipara	67 (59.9%)	137 (61.1)	204 (60.7%)
Grand multipara	36 (32.1%)	25 (11.2%)	61 (19.9%)
*Antenatal care*			
No follow-up	46 (41.1%)	11 (4.1%)	57 (17%)
Once	42 (37.5%)	36 (16.1%)	78 (23.2%)
Two or more	24 (21.4%)	177 (79%)	201 (59.8%)
*Gestational age*			
Less than 37 wks	6 (5.3%)	24 (10.7%)	30 (8.9%)
37–42 wks	99 (88.4%)	179 (79.9%)	278 (82.7%)
Greater than 42	2 (1.8%)	13 (5.8%)	15 (4.5)
Unknown	5 (4.5%)	8 (3.6%)	13 (3.9%)

Ninety-nine (88.4%) women from cases and 179 (79.9%) from control group were at term pregnancy.

**Table 2:** Classification of cases of uterine rupture based on their type occurred at Suhul General Hospital, Shire Town, North West Tigray, Ethiopia, 2016.

Classification of type of rupture	Frequency (%)
*Rupture based on the extent*	
Complete	93 (83.1%)
Incomplete	19 (16.9%)
*Site of uterine rupture*	
Anterior	107 (95.5%)
Posterior	13 (11.6%)
Left lateral	35 (3.1%)
Right lateral	11 (9.8%)
Lower segment	97 (86.6%)
Upper segment	9 (8%)

In classifying rupture based on the site, the sum exceeds the total case of ruptured uterus (112) since one rupture can involve tearing to multiple sites.

**Table 3:** Factors associated with uterine rupture at Shire General Hospital, North West Tigray, Ethiopia 2016.

Associated factors	Total samples (*n* = 336)		COR (CI)	*p* value	AOR (CI)
	Cases (*n* = 112)	Controls (*n* = 224)			
(1) Obstructed labor	69 (61.6%)	22 (9.8%)	14.73 (8.23–26.37)	0.00^∗^	5.76 (2.32–14.28)
(2) Malpresentation	39 (34.8%)	20 (8.9%)	5.45 (2.99–9.95)	0.00^∗^	4.48 (1.74–11.43)
(3) Paraity					
Nulipara^1^	9 (8.0%)	62 (27.7%)	—	0.00	—
Multipara	67 (59.8%)	137 (61.2%)	3.37 (1.58–7.18)	0.06	3.29 (0.95–11.34)
Grand multipara	36 (32.1%)	25 (11.2%)	9.92 (4.97–23.57)	0.02^∗^	6.32 (1.35–29.55)
(4) Interdelivery interval					
Less than 9 months^1^	14 (12.5%)	1 (0.4%)	—	0.00	—
9–18 months	46 (41.1%)	94 (41.9%)	0.35 (0.00–0.27) 0.04 (0.01–0.36)	0.21	6.80 (0.35–132.41)
Greater than 18 months	11 (9.8%)	19 (8.5%)	0.05 (0.01–0.38)	0.66	0.81 (0.32–2.05)
Unknown	32 (28.6%)	48 (21.4%)	—	0.22	0.35 (0.07–1.88)
(5) ANC follow-up					
No ANC	46 (41.1%)	11 (4.9%)	30.84 (14.02–67.54)	0.00^∗^	15.38 (4.55–46.99)
One ANC visit	42 (37.5%)	36 (16.1%)	8.60 (4.64–15.94)	0.00^∗^	4.62 (2.28–12.68)
Two or more^1^	24 (21.4%)	177 (79%)	—	0.00	—
(6) Duration of labor					
Less than 12 hours^1^	15 (13.4%)	155 (69.2%)	—	0.00	—
12–24 hours	27 (24.1%)	100 (44.6%)	2.07 (1.04–4.11)	0.18	1.88 (0.75–4.68)
Greater than 24 hr	70 (62.5%)	9 (4.0%)	59.63 (24.78–143.50)	0.00^∗^	24.39 (7.91–74.15)
(7) Onset of labor (spontaneous)	110 (98.2%)	200 (89.3%)	6.60 (1.53–28.45)	0.30	2.35 (0.46–11.95)

^∗^Significant variables, ^1^reference categories.

**Table 4:** Maternal and neonatal outcome of uterine rupture at Suhul General Hospital, Shire Town, North West Tigray, Ethiopia 2016.

Outcome	Case (*n* = 112)	Controls (*n* = 224)	Total 336
Fetal outcome			
Live birth with Apgar > 5	4 (3.4%)	212 (91.8%)	216 (62.0%)
Live birth with Apgar < 5	4 (3.4%)	5 (2.2%)	9 (2.5%)
Stillbirth	109 (93.1%)	14 (6.0%)	123 (35.3%)
Birth weight			
Less than 2500 gm	5 (4.3%)	19 (8.2%)	24 (6.9%)
2500–4000 gm	102 (87.1%)	207 (89.6%)	309 (88.8%)
Greater than 4000 gm	10 (8.5%)	5 (2.2%)	15 (4.3%)
Maternal outcome			
Stable	79 (70.5%)	214 (95.5%)	293 (87.2%)
Critical	29 (25.9%)	8 (3.6%)	37 (11%)
Referred	4 (3.6%)	1 (0.4%)	5 (1.5%)
Death	0 (0%)	1 (0.4%)	1 (0.3%)
Blood transfusion	35 (31.2%)	7 (3.1%)	42 (12.5%)
Operation done			
Repair of tear	61 (54.5%)	0	61 (18.1%)
TAH	41 (36.6%)	0	41 (12.2%)
STH	10 (8.9%)	0	10 (2.9%)
CS	0	16 (7.1%)	16 (4.7%)
BTL (*n* = 61)	47 (77%)	0	47 (13.9%)
Duration of hospitalization			
Less than 24 hr	0 (0)	145 (64.7%)	145 (43.1%)
24 hr-3 days	0 (0)	61 (27.2%)	61 (18.1%)
4–7 days	33 (29.4%)	14 (6.3%)	47 (14%)
8–12 days	62 (55.5%)	3 (1.3%)	65 (19.3%)
Greater than 12 days	17 (15.1%)	1 (0.4%)	18 (5.4%)

## References

[1] Turner M. J. (2002). Uterine rupture. *Best Practice & Research Clinical Obstetrics & Gynaecology*.

[2] Justus Hofmeyr G., Say L., Metin Gülmezoglu A. (2005). Systematic review: WHO systematic review of maternal mortality and morbidity: the prevalence of uterine rupture. *BJOG: An International Journal of Obstetrics & Gynaecology*.

[3] Al-Jufairi Z. A., Sandhu A. K., Al-Durazi K. A. (2001). Risk factors of uterine rupture. *Saudi Medical Journal*.

[4] Lydon-Rochelle M., Holt V. L., Easterling T. R., Martin D. P. (2001). Risk of uterine rupture during labor among women with a prior cesarean delivery. *New England Journal of Medicine*.

[5] Dow M., Wax J. R., Pinette M. G., Blackstone J., Cartin A. (2009). Third-trimester uterine rupture without previous cesarean: a case series and review of the literature. *American Journal of Perinatology*.

[6] Central statistics agency Ethiopia and ICF International: Ethiopia Demographic and Health Survey 2011, Addis Ababa Ethiopia and Calverton, Maryland, USA: Central statistical Agency and ICF International, 2012

[7] Berhan Y., Berhan A. (2014). Causes of maternal mortality in Ethiopia: a significant decline in abortion related death. *Ethiopian Journal of Health Sciences*.

[8] World Health Organization (2009). *Monitoring Emergency Obstetric Care: A Handbook*.

[9] Alemayehu W., Ballard K., Wright J. (2013). Primary repair of obstetric uterine rupture can be safely undertaken by non-specialist clinicians in rural Ethiopia: a case series of 386 women. *BJOG: An International Journal of Obstetrics & Gynaecology*.

[10] Chamiso B. (1995). Rupture of pregnant uterus in Shashemene General Hospital, south Shoa, Ethiopia (a three year study of 57 cases). *Ethiopian Medical Journal*.

[11] Wikipedia, the free encyclopedia; shirie town demographic profile 2007https://en.wikipedia.org/wiki/Shire Inda Selassie December, 2016

[12] Fitzpatrick K. E., Kurinczuk J. J., Alfirevic Z., Spark P., Brocklehurst P., Knight M. (2012). Uterine rupture by intended mode of delivery in the UK: a national case-control study. *PLoS Medicine*.

[13] Aboyeji A. P., Ijaiya M. D., Yahaya U. R. (2001). Ruptured uterus: a study of 100 consecutive cases in Ilorin, Nigeria. *Journal of Obstetrics and Gynaecology Research*.

[14] Admassu A. (2004). Analysis of ruptured uterus in Debre Markos Hospital, Ethiopia. *East African Medical Journal*.

[15] Kadowa I. (2010). Ruptured uterus in rural Uganda: prevalence, predisposing factors and outcomes. *Singapore Medical Journal*.

[16] Ezechi O. C., Mabayoje P., Obiesie L. O. (2004). Ruptured uterus in South Western Nigeria: a reappraisal. *Singapore Medical Journal*.

[17] Qudsia Q. A., Akhtar Z., Kamran K. H., Khan A. H. (2012). Woman health; uterus rupture, its complications and management in Teaching Hospital Bannu, Pakistan. *Maedica*.

[18] Omole-Ohonsi A., Attah R. (2011). Risk factors for ruptured uterus in a developing country. *Gynecology & Obstetrics*.

[19] Ofir K., Sheiner E., Levy A., Katz M., Mazor M. (2003). Uterine rupture: risk factors and pregnancy outcome. *American Journal of Obstetrics and Gynecology*.

[20] Adanu R. M., Obed S. A. (2003). Ruptured uterus: a seven-year review of cases from Accra, Ghana. *Journal of Obstetrics and Gynaecology Canada*.

[21] Latika S. A. (2006). 10 year analysis of uterine rupture at a teaching institution. *Journal of Obstetrics and Gynecology of India*.

[22] Yılmaz M., İsaoğlu Ü., Kadanalı S. (2011). The evaluation of uterine rupture in 61 Turkish pregnant women. *European Journal of General Medicine*.

[23] Gessessew A., Melese M. M. (2002). Ruptured uterus-eight year retrospective analysis of causes and management outcome in Adigrat Hospital, Tigray Region, Ethiopia. *Ethiopian Journal of Health Development*.

